# Role of Site-Specific N-Glycans Expressed on GluA2 in the Regulation of Cell Surface Expression of AMPA-Type Glutamate Receptors

**DOI:** 10.1371/journal.pone.0135644

**Published:** 2015-08-13

**Authors:** Yusuke Takeuchi, Jyoji Morise, Ippei Morita, Hiromu Takematsu, Shogo Oka

**Affiliations:** 1 Department of Biological Chemistry, Human Health Sciences, Graduate School of Medicine, Kyoto University, Kyoto, Japan; 2 Department of Biological Chemistry, Graduate School of Pharmaceutical Sciences, Kyoto University, Kyoto, Japan; Louisiana State University Health Sciences Center, UNITED STATES

## Abstract

The AMPA-type glutamate receptor (AMPAR), which is a tetrameric complex composed of four subunits (GluA1-4) with several combinations, mediates the majority of rapid excitatory synaptic transmissions in the nervous system. Cell surface expression levels of AMPAR modulate synaptic plasticity, which is considered one of the molecular bases for learning and memory formation. To date, a unique trisaccharide (HSO_3_-3GlcAβ1-3Galβ1-4GlcNAc), human natural killer-1 (HNK-1) carbohydrate, was found expressed specifically on N-linked glycans of GluA2 and regulated the cell surface expression of AMPAR and the spine maturation process. However, evidence that the HNK-1 epitope on N-glycans of GluA2 directly affects these phenomena is lacking. Moreover, it is thought that other N-glycans on GluA2 also have potential roles in the regulation of AMPAR functions. In the present study, using a series of mutants lacking potential N-glycosylation sites (N256, N370, N406, and N413) within GluA2, we demonstrated that the mutant lacking the N-glycan at N370 strongly suppressed the intracellular trafficking of GluA2 from the endoplasmic reticulum (ER) in HEK293 cells. Cell surface expression of GluA1, which is a major subunit of AMPAR in neurons, was also suppressed by co-expression of the GluA2 N370S mutant. The N370S mutant and wild-type GluA2 were co-immunoprecipitated with GluA1, suggesting that N370S was properly associated with GluA1. Moreover, we found that N413 was the main potential site of the HNK-1 epitope that promoted the interaction of GluA2 with N-cadherin, resulting in enhanced cell surface expression of GluA2. The HNK-1 epitope on N-glycan at the N413 of GluA2 was also involved in the cell surface expression of GluA1. Thus, our data suggested that site-specific N-glycans on GluA2 regulate the intracellular trafficking and cell surface expression of AMPAR.

## Introduction

Glycosylation is one of the major post-translational protein modifications with important roles in the structural and functional diversity of proteins. Among them, the human natural killer-1 (HNK-1) glyco-epitope is highly expressed on several cell adhesion molecules and extracellular matrix molecules in the nervous system [1]. This carbohydrate epitope, which exhibits a unique trisaccharide structure, (HSO_3_-3GlcAß1-3Galß1-4GlcNAc), is biosynthesized sequentially by galactosyltransferase (ß4GalT2) [2,3], one of two glucuronyltransferases (GlcAT-P and GlcAT-S) [4], and a sulfotransferase (HNK-1ST) [5]. We reported previously that GlcAT-P gene-deficient mice, which showed an almost complete loss of HNK-1 expression in the brain, exhibited an aberration in spatial learning and memory formation and a reduction of long-term potentiation in the hippocampal CA1 region [6]. These phenotypes might be due to abnormal dendritic spine morphogenesis [7]. Subsequently, we identified a candidate HNK-1-carrier protein, which is responsible for the defects in synaptic plasticity observed in GlcAT-P-deficient mice, as GluA2, a subunit of the AMPA-type glutamate receptor (AMPAR) [8].

**Fig 1:**
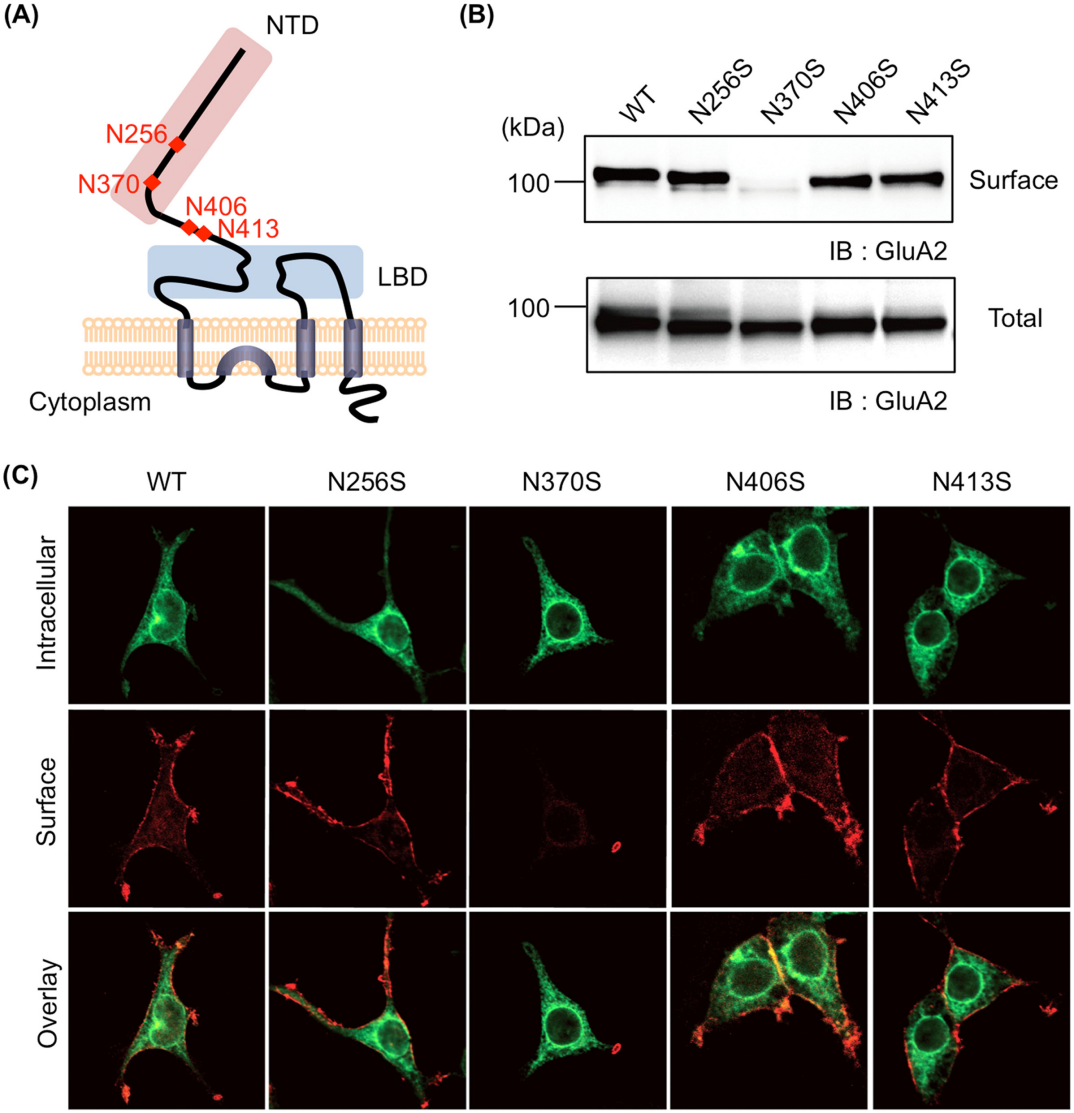
N-glycan at N370 is essential for cell surface expression of GluA2. (A) GluA2 is composed of NTD (pink), LBD (blue), transmembrane domains, and a cytoplasmic domain. NTD includes two N-glycosylation sites (N256 and N370), and N406 and N413 are located in the linker between NTD and LBD. The amino acid number was counted from the first methionine of the signal sequence. (B) A cell biotinylation assay was applied to HEK293 cells expressing GluA2 wild-type (WT) or N-glycosylation site mutants (N256S, N370S, N406S, or N413S). Biotinylated GluA2 was immunoblotted with anti-GluA2/3 polyclonal antibody (Surface). The lysates were also immunoblotted for loading control (Total). (C) HEK293 cells expressing WT or mutants were doubly immunostained. Cell surface GluA2 was stained with anti-GluA2 N-terminal monoclonal antibody (red) under nonpermeabilizing conditons. Intracellular GluA2 was subsequently stained with anti-GluA2/3 polyclonal antibody (green) after cell permeabilization.

AMPAR, one of the ionotropic glutamate receptors, a hetero- or homo-tetrameric complex composed of various combinations of four subunits (GluA1-4), mediates the majority of excitatory synaptic transmissions in the mammalian brain. Thus, the number of postsynaptic AMPARs contributes to long-lasting changes in synaptic strength and dendritic spine enlargement [9]. We previously showed that loss of the HNK-1 epitope greatly increases internalization of AMPARs in cultured hippocampal neurons and in heterologous cells, which indicates the HNK-1 epitope is an important factor in controlling the cell surface expression of the AMPAR [8]. However, as the HNK-1 epitope is expressed on several molecules, such as N-CAM, MAG, P0, and phosphacan [10,11], determining whether the HNK-1 epitope on GluA2 directly modifies cell surface expression of AMPAR is difficult. Moreover, GluA2 has four potential N-glycosylation sites in its extracellular domain (Fig 1A). Therefore, questions regarding the particular N-glycosylation sites on GluA2 that dominantly possess the HNK-1 epitope and whether other N-glycans have a role in regulating the cell surface expression of GluA2 remain unanswered.

In the present study, we generated mutants in the potential GluA2 N-glycosylation sites (N256S, N370S, N406S, and N413S) to demonstrate the roles of N-glycans, including the HNK-1 epitope, in regulating the cell surface expression of GluA2. We demonstrated that N-glycan at N370 was involved in the intracellular trafficking of GluA2 and co-transfected GluA1. We also found that the HNK-1 epitope was mainly expressed on N-glycan at N413 and that the HNK-1 epitope on GluA2 regulated the cell surface expression of co-transfected GluA1. Taken together, our results demonstrated that the site-specific N-glycosylation of GluA2 (the HNK-1 epitope at N413 and N-glycan at N370) is required for the intracellular trafficking and cell surface expression of GluA1 and GluA2.

## Materials and Methods

### Expression Plasmids

To yield the plasmid pcDNA3.1/GluA1, the EcoRI-EcoRI GluA1 fragment derived from pKC24/GluA1 (mouse), donated by Dr. M. Mishina (Tokyo University) [12], was cloned into pcDNA3.1/myc-HisB (Invitrogen). Constructs of the plasmid pcDNA3.1/GluA2 and pIRES/GlcAT-P-IRES-HNK-1ST were described previously [8,13]. A series of GluA2 mutants in which the asparagine (Asn) residue in the consensus sequence (N-X-S/T) was mutated to serine (Ser) (N256S, N370S, N406S, and N413S) were constructed using QuickChange Lightning site-directed mutagenesis kit (Stratagene) with the following primers: (N256S; cagtttggaggagcaagtgtctctggatttcag, N370S; ccagaacggaaaacgaataagctacacaattaacatcatgg, N406S; agctcccctctggaagtgacacatctgggct, N413S; catctgggcttgaatcaaaaactgtggttgtcacc; and the respective reverse complementary primers), employing pcDNA3.1/GluA2 as a template. The pcDNA3.1/N-cadherin was a generous gift from Dr. A. Kinoshita (Kyoto University) [14].

### Cell Culture and Transfection

HEK293 cells purchased from the American Type Culture Collection were cultured in Dulbecco’s modified Eagle’s medium supplemented with 10% fetal calf serum at 37°C until reaching 50–80% confluency. For transfection, cells were plated on 60-mm tissue culture dishes or four-well chamber slides, grown overnight, and then transfected with various expression plasmids (2 μg/dish) using FuGENE6 transfection reagent (Roche Applied Science).

### Cell Surface Protein Biotinylation Assay

At 48 h post-transfection, HEK293 cells were treated with 1 mg/ml EZ-Link Sulfo-NHS-SS-biotin (Thermo Fisher Scientific) in phosphate-buffered saline (PBS) for 30 min at 4°C. The cells were solubilized with lysis buffer (1% TritonX-100, 0.5% deoxycholate, 20 mM Tris-HCl (pH7.4), 150 mM NaCl, 1 mM EDTA, and protease inhibitor mixture). Cell surface-biotinylated proteins were pulled down with Immobilized Streptavidin Agarose Resin (Thermo Fisher Scientific).

### Immunoprecipitation

At 48 h post-transfection, HEK293 cells were solubilized with lysis buffer. For immunoprecipitation, primary antibodies (final concentration 4 μg/ml) and Protein G-Sepharose 4 Fast Flow (GE Healthcare) were added to the solution and rotated for 2 h at 4°C.

### SDS-PAGE and Immunoblotting

Biotinylated or immunoprecipitated proteins and cell lysates were separated using 7% sodium dodecyl sulfate-polyacrylamide gel electrophoresis (SDS-PAGE) with the Laemmli buffer system and then transferred to nitrocellulose membranes. After blocking with 5% nonfat dried milk in PBS containing 0.05% Tween 20, the membranes were incubated with specific primary antibodies (final concentration 1 μg/ml) followed by appropriate horseradish peroxidase (HRP)-conjugated secondary antibodies (final concentration 1 μg/ml). Proteins were then detected with SuperSignal West Pico Chemiluminescent Substrate (Thermo Fisher Scientific) using a Luminoimage Analyzer LAS-3000 (FujiPhoto Film). The antibodies used in the immunoblotting (or immunoprecipitation) were as follows: HNK-1 monoclonal antibody (a hybridoma cell line was purchased from the American Type Culture Collection), anti-GluA1 polyclonal antibody (Enzo Life Sciences), anti-GluA2/3 polyclonal antibody (Enzo Life Sciences), anti-N-cadherin monoclonal antibody (BD Biosciences). Each immunoblotting experiment was replicated two or three times, and representative figures are shown for each experiment.

### Immunofluorescence Staining

At 36 h post-transfection, HEK293 cells were washed with PBS. To detect GluA2 on the cell surface membrane, cells were fixed in 4% paraformaldehyde in PBS for 10 min at room temperature and incubated with anti-GluA2 N-terminus monoclonal antibody (Millipore, final concentration 5 μg/ml) in PBS containing 3% bovine serum albumin (BSA) for 1 h at room temperature (nonpermeabilizing conditions). To detect the intracellular GluA2, the cells were then fixed in methanol for 15 min at -20°C and incubated with anti-GluA2/3 polyclonal antibody (Enzo Life Sciences, final concentration 5 μg/ml) in PBS containing 3% BSA for 1 h at room temperature (permeabilizing conditions) followed by incubation with AlexaFluor488-conjugated anti-rabbit IgG antibody and AlexaFluor546-conjugated anti-mouse IgG antibody (Molecular Probes, final concentration 5 μg/ml) in PBS containing 3% BSA for 1 h at room temperature. Intracellular and cell surface GluA2 were observed using the FluoView imaging system (Olympus).

## Results

### Loss of N-glycan at N370 reduced cell surface expression of GluA2

We previously determined that the HNK-1 epitope was attached to the non-reducing terminus of N-glycans on GluA2 [8], but it remains unclear at which N-glycosylation site(s) the HNK-1 epitope is expressed. To investigate the N-glycosylation site(s) of HNK-1 on GluA2, we first constructed mutants lacking each N-glycan on GluA2 by replacing the Asn residues of four potential N-glycosylation sites with Ser (N256S, N370S, N406S, and N413S) (Fig 1A). The wild-type (WT) and mutants were transfected into HEK293 cells, and the surface expression level was examined using a biotinylation assay. We found the cell surface expression of N370S was almost completely abolished but equivalent to WT in the other mutants (Fig 1B). Next, to investigate the subcellular localization of WT and mutants, immunofluorescence staining was initially performed under nonpermeabilizing conditions to detect the cell surface GluA2; it was subsequently performed under permeabilizing conditions to detect the intracellular GluA2. Almost all N370S were distributed primarily in intracellular sites, which may represent the endoplasmic reticulum (ER) (Fig 1C). However, other mutants and WT showed both cell surface and intracellular localizations. These results indicated that, of the four potential N-glycosylation sites, the N-glycan at N370 strongly affected the cell surface expression of GluA2.

### HNK-1 epitope dominantly expressed on N-glycan at N413

**Fig 2:**
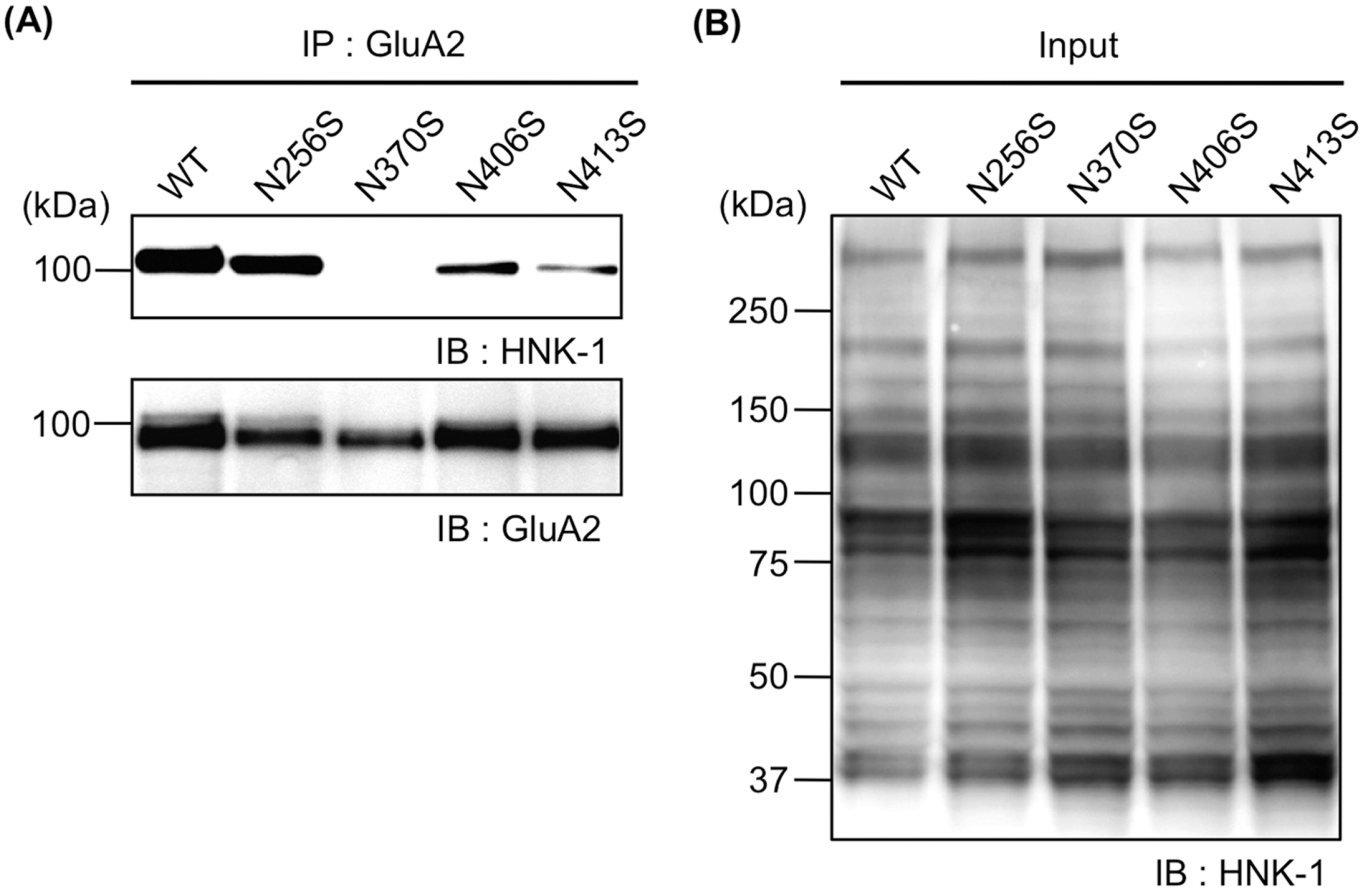
The HNK-1 epitope is preferentially expressed on N-glycan at N413. GluA2 (WT, N256S, N370S, N406S, or N413S) was transfected into HEK293 cells with HNK-1-synthesizing enzymes (GlcAT-P and HNK-1ST). After immunoprecipitation (IP) with anti-GluA2/3 polyclonal antibody, immunoprecipitates were immunoblotted with an HNK-1 monoclonal antibody and anti-GluA2/3 polyclonal antibody (A). The cell lysates were immunoblotted with an HNK-1 monoclonal antibody (Input) (B).

To investigate the HNK-1 expression sites on GluA2, we transfected WT or N-glycosylation site mutants with HNK-1-synthesizing enzymes (GlcAT-P and HNK-1ST) into HEK293 cells and immunoprecipitated GluA2 with anti-GluA2/3 polyclonal antibodies. Then, the HNK-1 expression level on each mutant was analyzed with an HNK-1 monoclonal antibody. When the HNK-1 and GluA2 expression levels on each cell lysate were almost equal (Fig 2A, lower panel and Fig 2B), the HNK-1 expression level on N256S was similar to that on WT, whereas the HNK-1 expression on N370S was almost completely abolished. Additionally, N406S and N413S exhibited modest reductions in HNK-1 expression, respectively (Fig 2A, upper panel). This result suggested the HNK-1 epitope was not equally expressed on all four N-glycosylation sites but was expressed on limited sites. N370S, which was not transported from the ER to the Golgi apparatus (Fig 1), showed no HNK-1 expression, because the GlcAT-P responsible for the expression of HNK-1 localizes in the Golgi apparatus.

### HNK-1 epitope at N413 enhanced cell surface expression of GluA2

**Fig 3:**
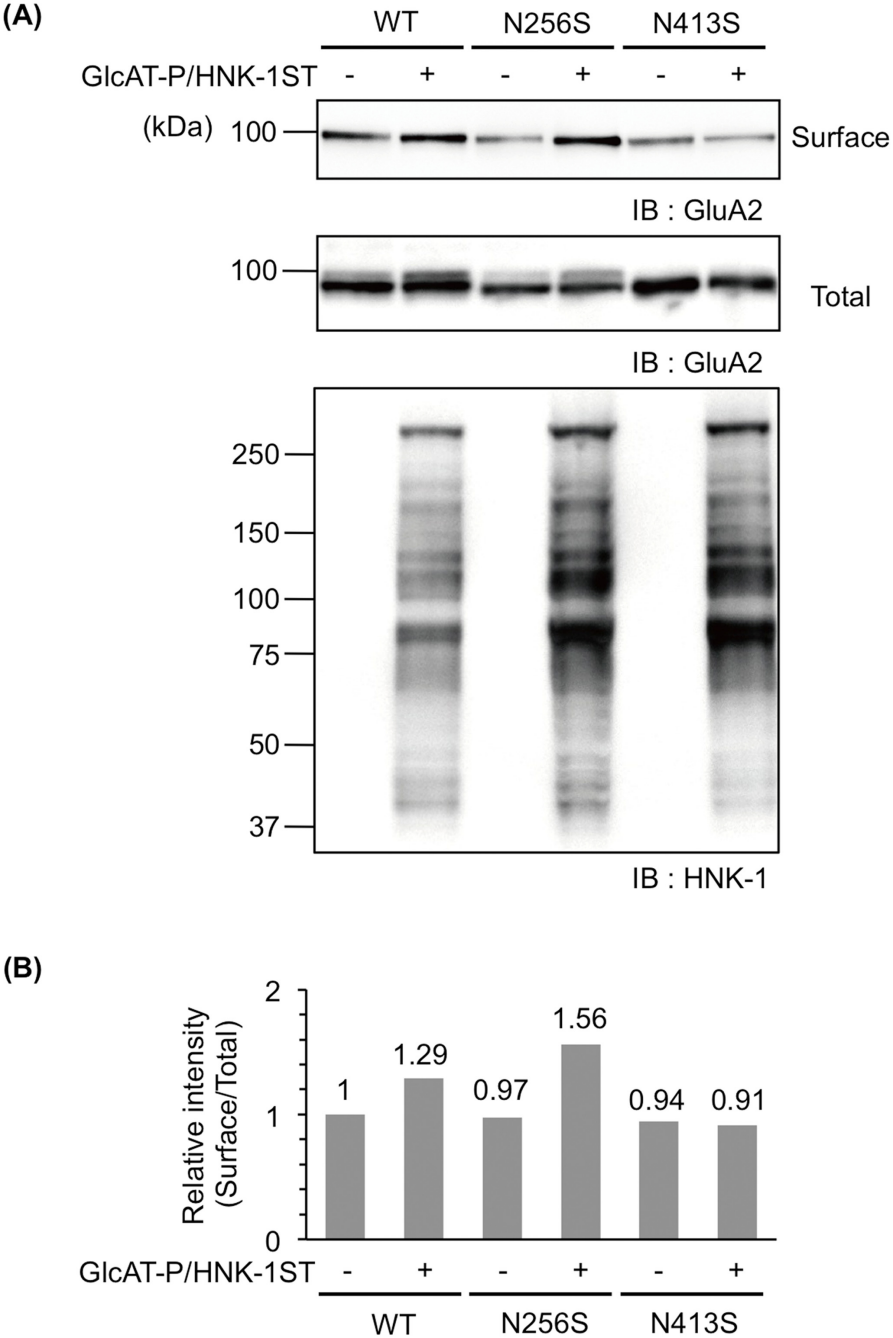
The HNK-1 epitope on N-glycan at N413 enhances cell surface expression of GluA2. (A) A cell biotinylation assay was applied to HEK293 cells expressing GluA2 (WT, N256S, or N413S) with (+) or without (-) HNK-1-synthesizing enzymes (GlcAT-P and HNK-1ST). Biotinylated GluA2 was immunoblotted with anti-GluA2/3 polyclonal antibody (Surface). The cell lysates were also immunoblotted with the same polyclonal antibody (Total) and an HNK-1 monoclonal antibody. (B) Relative intensities of surface expression levels of GluA2 (surface/total) were calculated and normalized with that of WT without the HNK-1 epitope.

As the HNK-1 epitope is widely expressed on a series of cell adhesion molecules and extracellular molecules other than GluA2 [1], whether the HNK-1 epitope on GluA2 directly regulates the cell surface expression of GluA2 is unclear. To investigate this issue, we used N413S because the expression of HNK-1 was the most reduced (Fig 2). Additionally, we used N256S as a control because this mutant showed similar HNK-1 expression as WT. These mutants and WT were transfected with or without HNK-1-synthesizing enzymes into HEK293 cells and a cell surface biotinylation assay was applied. Consistent with our previous report [8], the expression of the HNK-1 epitope increased the cell surface expression levels of WT and N256S (Fig 3A, upper panel and Fig 3B). However, in the case of the N413S mutant, the cell surface expression level was not increased by HNK-1 epitope expression (Fig 3A, upper panel and Fig 3B). Considering that the total HNK-1 expression level of the N413S-transfected cells was similar to N256S (Fig 3A, lower panel), this result indicated that the HNK-1 epitope on GluA2 (especially at N413) was required for enhancing the cell surface expression.

### HNK-1 epitope at N413 enhanced the interaction of GluA2 with N-cadherin

**Fig 4:**
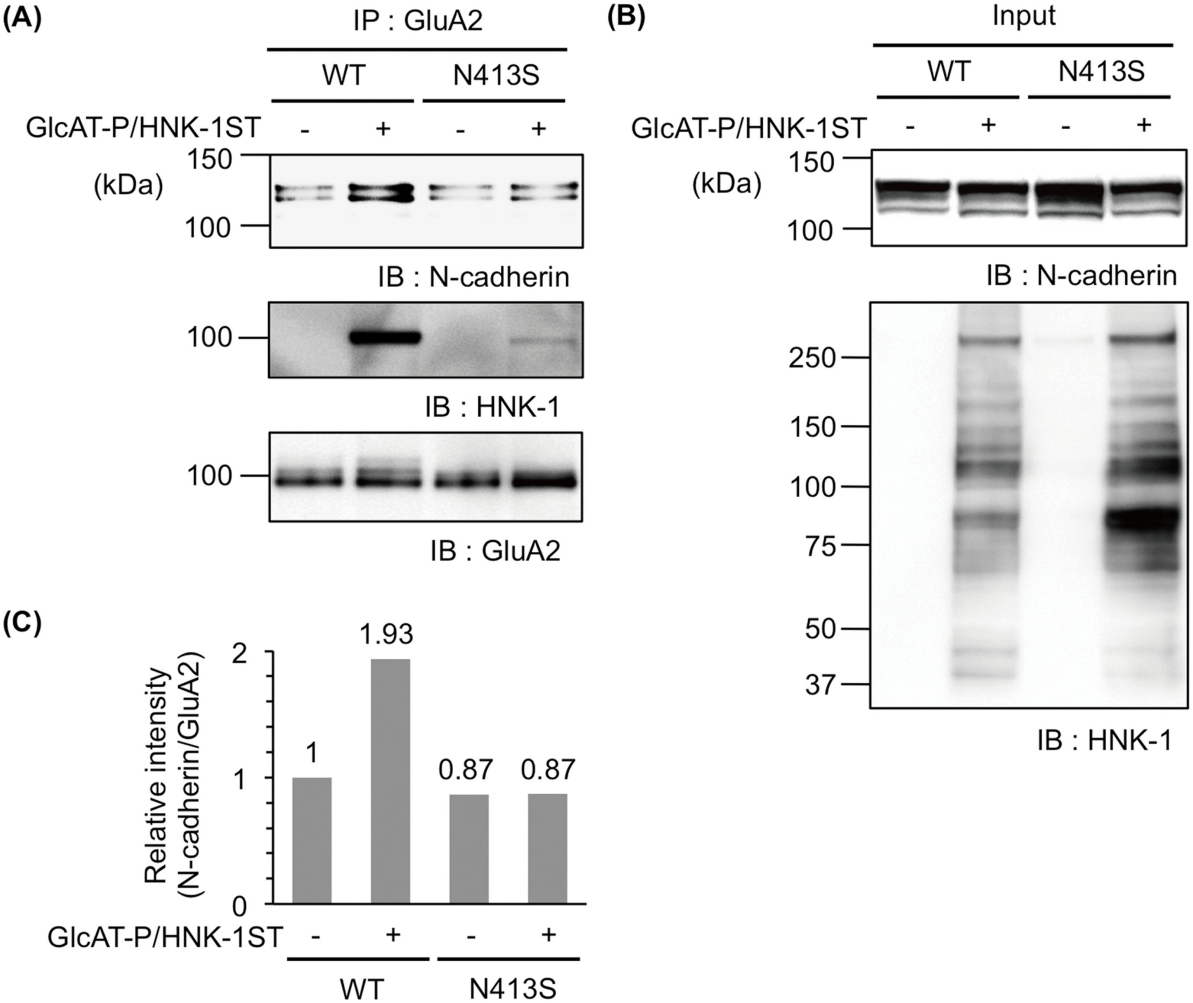
The HNK-1 epitope on N-glycan at N413 promotes interaction with N-cadherin. N-cadherin and GluA2 (WT or N413S) were transfected into HEK293 cells with (+) or without (-) HNK-1-synthesizing enzymes (GlcAT-P and HNK-1ST). After immunoprecipitation (IP) with anti-GluA2/3 polyclonal antibody, immunoprecipitates were immunoblotted with anti-N-cadherin and HNK-1 monoclonal antibodies and anti-GluA2/3 polyclonal antibody (A). The cell lysates were immunoblotted for loading control (Input) (B). (C) Relative intensities of co-immunoprecipitated N-cadherin levels (N-cadherin/GluA2) were calculated and normalized with that of WT without the HNK-1 epitope.

GluA2 interacts with N-cadherin *via* the extracellular N-terminal domain, and this interaction regulates the cell surface diffusion, intracellular trafficking, and cell surface expression of AMPAR on neuronal membranes [15,16]. Moreover, we previously demonstrated that the interaction between GluA2 and N-cadherin was regulated in an HNK-1-dependent manner in GlcAT-P-deficient mice and heterologous cells [8]. However, it remains unclear whether the HNK-1 epitope expressed on N-glycan of GluA2 is responsible for enhancing the interaction of GluA2 with N-cadherin. Therefore, we transfected WT or N413S and N-cadherin with or without HNK-1-synthesizing enzymes. After immunoprecipitation with anti-GluA2/3 polyclonal antibody, N-cadherin was immunoblotted to compare the amount of N-cadherin associated with GluA2. Consistent with our previous report [8], HNK-1 expression increased the amount of N-cadherin co-immunoprecipitated with WT (Fig 4A, upper panel and Fig 4C). However, HNK-1 expression had a minimal effect on the amount of N-cadherin co-immunoprecipitated with N413S (Fig 4A, upper panel and Fig 4C). Although the total HNK-1 expression level of N413S-expressing cells was higher than that in WT, the HNK-1 epitope expression in N413S was lower than that in WT (Fig 4B, lower panel and Fig 4A, middle panel). Therefore, other HNK-1-carrier proteins had little or no effect on the interaction of GluA2 with N-cadherin, suggesting that the HNK-1 epitope on N-glycan at N413 has a major role in enhancing the interaction with N-cadherin.

### Loss of N-glycan at N370 downregulated the intracellular trafficking of GluA1

**Fig 5:**
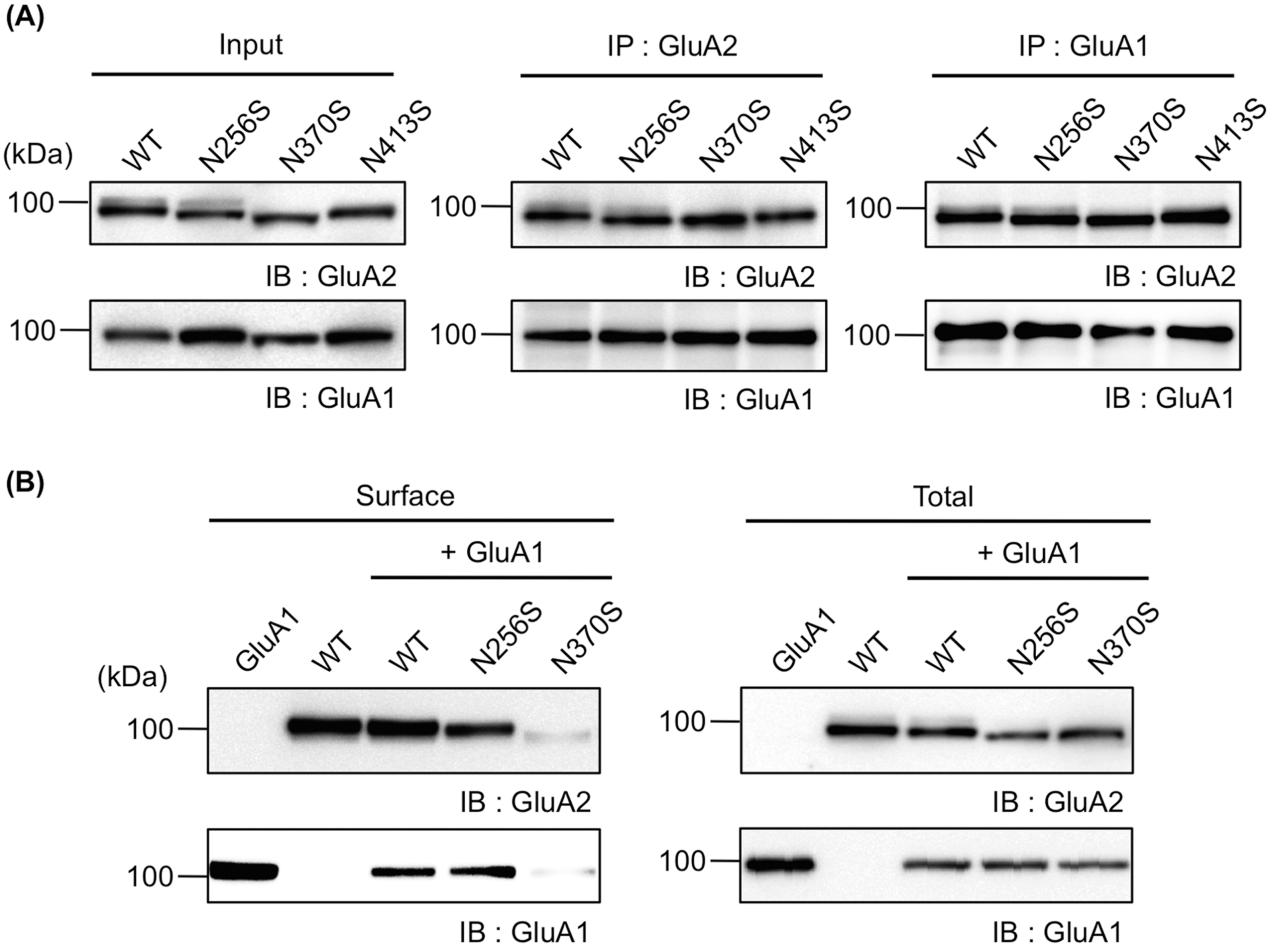
N-glycan at N370 of GluA2 regulates the intracellular trafficking of GluA1. (A) GluA1 and GluA2 (WT, N256S, N370S, or N413S) were transfected into HEK293 cells. GluA1 and GluA2 were immunoprecipitated with anti-GluA1 (right) and anti-GluA2/3 (middle) polyclonal antibodies, respectively, and then immunoblotted with each antibody. The cell lysates were also immunoblotted for loading control (Input) (left). (B) A cell biotinylation assay was applied to HEK293 cells expressing GluA1 and/or GluA2 under several conditions: GluA1 alone, GluA2WT alone, GluA1 and GluA2WT, GluA1 and GluA2N256S, or GluA1 and GluA2N370S. Biotinylated GluA1 and GluA2 were immunoblotted with anti-GluA1 and anti-GluA2/3 polyclonal antibodies, respectively (Surface). The cell lysates were also immunoblotted for loading control (Total).

The majority of AMPARs in the nervous system function as hetero-tetramers composed of GluA1 and GluA2 [17]. There is a distinct trafficking mode in two subunits; GluA1 shows constitutive trafficking from the ER to the Golgi, whereas GluA2 is retained in the ER and shows slow forward trafficking in hippocampal neurons [18,19]. However, the identity of the subunits that dictate the AMPAR exiting the ER and the issue of whether N-glycosylation on GluA2 affects the trafficking of GluA1 remain unclear. To investigate how the exiting and intracellular trafficking are governed by N-glycosylation on GluA2, we first examined whether the N-glycosylation site mutants of GluA2 could associate with GluA1. Thus, GluA2WT or mutants (GluA2N256S, GluA2N370S, and GluA2N413S) were transfected with GluA1 into HEK293 cells and GluA1 or GluA2 was immunoprecipitated with anti-GluA1 or anti-GluA2/3 polyclonal antibodies, respectively. Then, the amount of co-immunoprecipitation was evaluated using immunoblotting analysis with each antibody. GluA1 equally immunoprecipitated with GluA2WT and each mutant and *vice versa* (Fig 5A), indicating each mutant could associate with GluA1 as well as GluA2WT.

**Fig 6:**
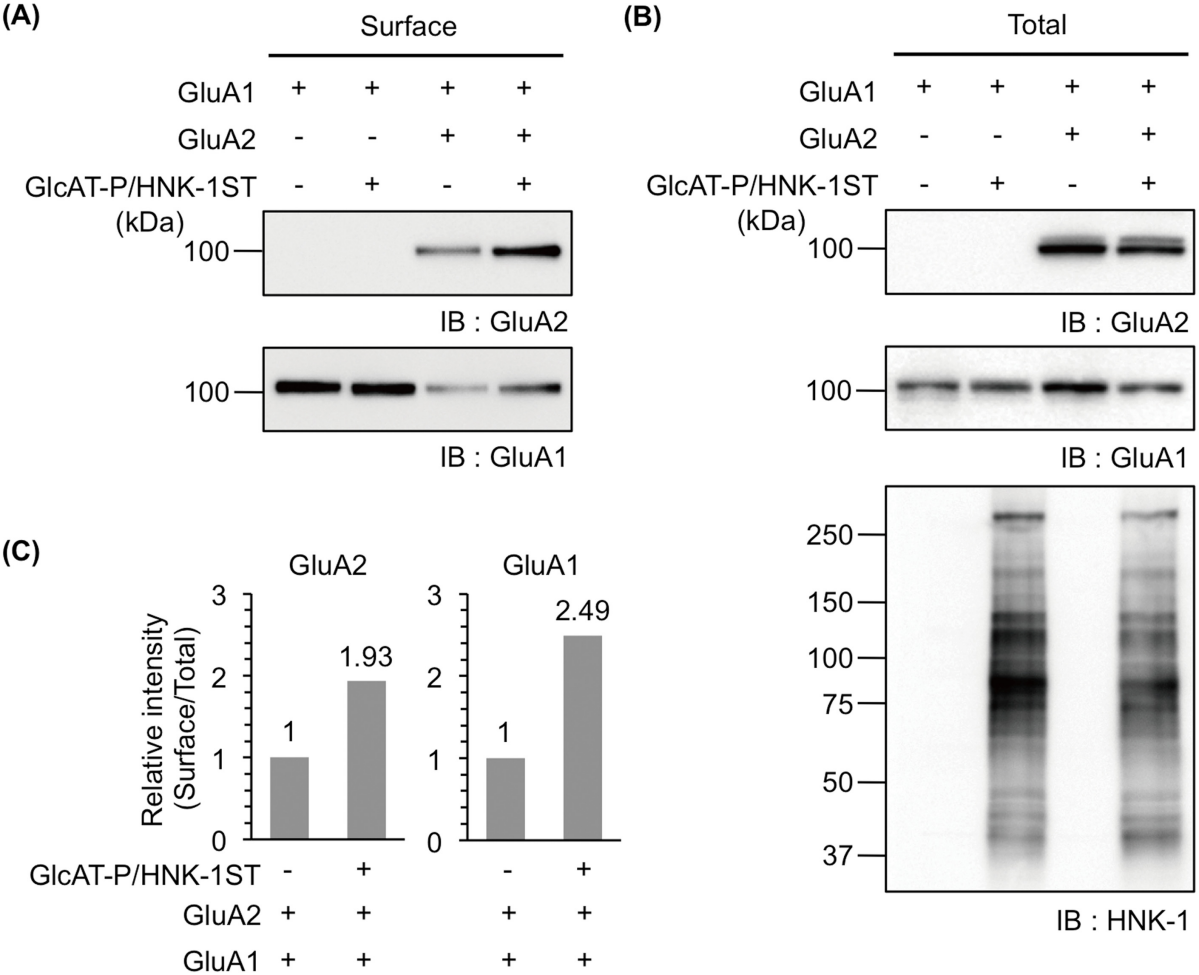
The HNK-1 epitope expressed on GluA2 enhances cell surface expression of GluA1. A cell biotinylation assay was applied to HEK293 cells expressing GluA1 with (+) or without (-) GluA2 or HNK-1-synthesizing enzymes (GlcAT-P and HNK-1ST) as indicated. Biotinylated GluA1 and GluA2 were immunoblotted with anti-GluA1 and anti-GluA2/3 polyclonal antibodies, respectively (Surface) (A). The cell lysates were also immunoblotted with the same polyclonal antibodies (Total) and an HNK-1 monoclonal antibody (B). (C) Relative intensities of surface expression levels of GluA2 or GluA1 (surface/total) were calculated and normalized with or without the HNK-1 epitope.

Next, to analyze the effect of N-glycosylation, especially N-glycan at N370, on the trafficking of GluA1, GluA1 and GluA2 (GluA2WT, GluA2N256S, or GluA2N370S) was co-transfected into HEK293 cells, and each cell surface expression level was evaluated using a cell surface biotinylation assay. The cell surface expression of GluA1 appeared to be downregulated by co-expression of GluA2WT and GluA2N256S (Fig 5B, left lower panel). This propensity is also shown in Fig 6A. In contrast, GluA2WT and GluA2N256S were not altered by GluA1 expression. (Fig 5B, left upper panel). This suggested that the forward trafficking of GluA1 might be controlled by GluA2. Moreover, the cell surface expression of GluA1 was prominently downregulated by co-expressing GluA2N370S (Fig 5B, left lower panel). In addition, the reduced cell surface level of GluA2N370S remained evident even when GluA1 was co-expressed (Fig 5B, left upper panel). These results suggest that GluA2 controlled the intracellular trafficking of GluA1 in HEK293 cells and that N-glycan at N370 of GluA2 was responsible for this trafficking.

### HNK-1 epitope on GluA2 enhances cell surface expression of GluA1

We previously showed that the HNK-1 epitope was specifically expressed on GluA2 but not on GluA1 *in vivo* and that it was involved in the cell surface expression of GluA2 [8]. In the present study, we showed that the HNK-1 epitope on GluA2 but not on other proteins is essential for enhancing the cell surface expression of GluA2 (Fig 3). Moreover, N-glycan at N370 of GluA2 is necessary for the intracellular trafficking of GluA2 and GluA1 (Fig 5). Based on these results, we hypothesized that the HNK-1 epitope on GluA2 also regulates the cell surface level of GluA1. Therefore, we transfected GluA1 or GluA2 with or without HNK-1-synthesizing enzymes into HEK293 cells. Next, we performed the cell surface biotinylation assay. Consistent with Fig 5B, the cell surface expression of GluA1 was reduced by co-expression with GluA2 (Fig 6A, lower panel). Under these conditions, the HNK-1 expression increased the cell surface expression of GluA1 as well as GluA2 (Fig 6A, upper and lower panels and Fig 6C). The HNK-1 epitope did not affect the cells expressing GluA1 alone (Fig 6A, lower panel); thus, the HNK-1 epitope on GluA2 regulated the cell surface expression of GluA1. These results suggest the HNK-1 epitope on GluA2 could be involved in the cell surface expression of the AMPAR complex formed by GluA1 and GluA2.

## Discussion

GlcAT-P-deficient mice lacking almost all the HNK-1 epitope in the brain showed aberrations in synaptic plasticity [6]. This might be due to the immature spine morphology responsible for the reduction in the cell surface expression of AMPAR [7]. We previously demonstrated that the HNK-1 epitope was expressed on the N-glycan nonreducing terminal of GluA2, the subunit of which has four potential N-glycosylation sites [8]. The HNK-1 expression increased the interaction of GluA2 with N-cadherin, a binding partner of GluA2, resulting in the stable cell surface expression of AMPAR. Thus, the HNK-1 epitope plays important roles in modulating synaptic plasticity. However, evidence that the HNK-1 epitope on GluA2 directly regulates the neuronal functions including the synaptic plasticity is lacking because the involvement of the HNK-1 epitope on other molecules cannot be excluded. In the present study using the N-glycosylation site mutants of GluA2, we showed that N413 of GluA2 was the major potential site of the HNK-1 epitope (Fig 2). Furthermore, the HNK-1 epitope at N413 directly regulated the cell surface expression of AMPAR and the interaction with N-cadherin (Figs 3, 4 and 6). It is considered that the up-regulation of cell surface expression of GluA2 caused by the HNK-1 epitope at N413 (Fig 3) may be attributed to the enhancement of the interaction with N-cadherin (Fig 4). Therefore, the reduction in the cell surface expression of AMPAR observed in GlcAT-P-deficient mice was probably responsible for the loss of the HNK-1 epitope on GluA2 but not on other HNK-1-carrier proteins.

N-glycosylation sites of N406 and N413 are located on the linker region between the N-terminal domain (NTD) and the ligand-binding domain (LBD) (Fig 1A). A previous report showed that N-glycans located on the linker region of GluA4 did not influence cell surface expression of GluA4 in HEK293 cells [20]. This is in agreement with our results (Fig 1) showing the cell surface expression level of N406S and N413S mutants was similar to that of WT in HEK293 cells. However, we demonstrated that N-glycan at N413 of GluA2 was important for cell surface expression when the HNK-1 epitope was expressed (Figs 3 and 6). Although we did not examine whether the HNK-1 epitope was expressed on GluA4 *in vivo*, the extracellular domain of GluA4 were more similar to GluA2 than to GluA1, suggesting the HNK-1 epitope could be expressed on GluA4 and may control cell surface expression.

Reportedly, the region responsible for the interaction between N-cadherin and GluA2 is that of the first 92 N-terminal amino acids of the extracellular domain of GluA2 [15], but the N-cadherin region involved in this interaction remains undetermined. In this study, we showed that the HNK-1 epitope on N413 of GluA2 enhanced the interaction with N-cadherin (Fig 4). However, it is unlikely that the HNK-1 epitope on N413 regulates the interaction with N-cadherin through the 92 amino acids because N413 is distant from the N-terminal region. Potentially, a different domain on N-cadherin would interact with an HNK-1 epitope other than the 92 N-terminal amino acids. Another possibility is that N-glycan at the N413 of one subunit may affect the interaction of the 1–92 region of a neighboring subunit because AMPAR is present as a tetramer. We cannot currently exclude this possibility. In any case, it is important to identify the N-cadherin regions participating in these interactions.

Of the four potential N-glycosylation sites, the N370S mutant showed almost complete reduction in cell surface expression (Fig 1). We also analyzed its glycosylation pattern and found N370S had only high-mannose type N-glycan (unpublished observation), which is a characteristic of glycoproteins in the ER indicating that N370S exists primarily in the ER. Therefore, N-glycan at N370 of GluA2 is crucial for the intracellular trafficking of GluA2 from the ER to the Golgi. Currently, the reason that GluA2 lacking N-glycan at N370 was retained in the ER remains unclear. Possibly, the N370S was trapped in the ER as a misfolded protein by quality control systems. However, because N370S interacted with GluA1 (Fig 5A) and suppressed the cell surface expression of GluA1 (Fig 5B), the N-glycan at N370 may be directly involved in intracellular forward trafficking but not with GluA2 protein folding. Therefore, the N-glycan at N370 might be directly involved in intracellular forward trafficking but not in GluA2 protein folding.

Because most AMPARs in neurons exert their function as hetero-tetramers GluA1/GluA2 [17], the trafficking mode has been studied, but the subunits that regulate exiting the ER remain unclear. For example, depending on neuronal activity, the trafficking of GluA1/GluA2 to the cell surface membrane is dictated by the GluA1 C-terminus [21]. Conversely, the GluA2 C-terminus is also an important factor for the trafficking of GluA2 from the ER when Ca^2+^ is released from the ER following Ca^2+^/Calmodulin-activated kinase II (CAMKII) activation [22]. However, in this study, we showed the cell surface expression of GluA1 was suppressed by co-expression with GluA2WT and reduced in the presence of GluA2N370S (Fig 5). Therefore, the intracellular trafficking of GluA1 is considered constitutively controlled by GluA2 and by its N-glycan at N370, at least in HEK293 cells. Examining whether this trafficking mode is also formed in neurons or whether N-glycans on GluA2 actually dictates the trafficking of GluA1 *in vivo* is important. In this respect, GluA2 used in this study is likely unsuitable for the experiment using primary cultured neurons, because it is difficult to distinguish between endogenously expressed GluA2 and transfected GluA2. The development of suitable epitope-tagged GluA2 mutants would also enable us to demonstrate that the HNK-1 epitope on GluA2 directly functions in spine formation.

GluA2 controls the critical biophysical properties of the AMPAR and plays pivotal roles in forming long-term synaptic plasticity [23]. Previously, GluA2 was shown to interact with its intracellular and extracellular binding partners, but the role of glycosylation in the interaction has not been studied. Herein, we showed that site-specific glycosylation of GluA2 (N-glycan at N370 and the HNK-1 epitope on N-glycan at N413) was involved in the intracellular trafficking and cell surface expression of AMPAR. Overall, our present report may help elucidate additional AMPAR functions, such as interactions with other proteins, trafficking of other subunits, and roles in synaptic plasticity.
